# Evaluating the impact of a falls prevention community of practice in a residential aged care setting: a realist approach

**DOI:** 10.1186/s12913-017-2790-2

**Published:** 2018-01-15

**Authors:** Jacqueline Francis-Coad, Christopher Etherton-Beer, Caroline Bulsara, Nicole Blackburn, Paola Chivers, Anne-Marie Hill

**Affiliations:** 1 School of Physiotherapy, Institute for Health Research, The University of Notre Dame Australia, 19 Mouat St, Fremantle, Western Australia 6959 Australia; 2 School of Medicine and Pharmacology, The University of Western Australia, 35 Stirling Hwy, Crawley, Western Australia 6009 Australia; 3 School of Nursing and Midwifery, The University of Notre Dame Australia, 19 Mouat St, Fremantle, Western Australia 6959 Australia; 4 Brightwater Group, 355 Scarborough Beach Rd, Osborne Park, Western Australia 6017 Australia; 5 Institute for Health Research, The University of Notre Dame Australia, 19 Mouat St, Fremantle, Western Australia 6959 Australia; 6 School of Physiotherapy and Exercise Science, Curtin University, GPO Box U1987, Perth, Western Australia 6845 Australia

**Keywords:** Community of practice, Falls prevention, Realist approach, Evaluation, Translation, Residential aged care

## Abstract

**Background:**

Falls are a major socio-economic problem among residential aged care (RAC) populations resulting in high rates of injury including hip fracture. Guidelines recommend that multifactorial prevention strategies are implemented but these require translation into clinical practice. A community of practice (CoP) was selected as a suitable model to support translation of the best available evidence into practice, as it could bring together like-minded people with falls expertise and local clinical knowledge providing a social learning opportunity in the pursuit of a common goal; falls prevention. The aims of this study were to evaluate the impact of a falls prevention CoP on its membership; actions at facility level; and actions at organisation level in translating falls prevention evidence into practice.

**Methods:**

A convergent, parallel mixed methods evaluation design based on a realist approach using surveys, audits, observations and semi-structured interviews. Participants were 20 interdisciplinary staff nominating as CoP members between Nov 2013-Nov 2015 representing 13 facilities (approximately 780 beds) of a RAC organisation. The impact of the CoP was evaluated at three levels to identify how the CoP influenced the observed outcomes in the varying contexts of its membership (level i.), the RAC facility (level ii.) and RAC organisation (level iii.).

**Results:**

Staff participating as CoP members gained knowledge and awareness in falls prevention (*p* < 0.001) through connecting and sharing. Strategies prioritised and addressed at RAC facility level culminated in an increase in the proportion of residents supplemented with vitamin D (*p* = 0.002) and development of falls prevention education. At organisation level a falls policy reflecting preventative evidence-based guidelines and a new falls risk assessment procedure with aligned management plans were written, modified and implemented. A key disenabling mechanism identified by CoP members was limited time to engage in translation of evidence into practice whilst enabling mechanisms included proactive behaviours by staff and management.

**Conclusions:**

Interdisciplinary staff participating in a falls prevention CoP gained connectivity and knowledge and were able to facilitate the translation of falls prevention evidence into practice in the context of their RAC facility and RAC organisation. Support from RAC organisational and facility management to make the necessary investment in staff time to enable change in falls prevention practice is essential for success.

**Electronic supplementary material:**

The online version of this article (10.1186/s12913-017-2790-2) contains supplementary material, which is available to authorized users.

## Background

Falls are a major socio-economic problem in the residential aged care (RAC) sector; half its population fall annually [1–3] and 25–30% of these falls result in physical injury [3–5]. Consequences for residents who fall include increased risk of mortality, functional decline, depression and anxiety [4, 6, 7] in addition to significant cost burden for the health sector [8, 9]. Preventing falls and resultant injury is challenging due to the multifactorial nature of falls, the complex characteristics of RAC populations who have multiple co-morbidities with age-related systems decline [5, 10, 11] and a diversely skilled workforce caring for them [10, 12]. Two recent meta analyses in RAC populations showed different findings; the Cochrane review [13] found supplementing residents with low vitamin D levels reduced the rate of falls by 37% but not the risk of falling whilst Vlaeyen et al. [7] reported multifactorial interventions delivered by a multidisciplinary staff reduced falls by 33% and the number of recurrent fallers by 21%. Falls prevention evidence based guidelines also offer strategy implementation and adoption advice at staff, facility and organisation levels [14, 15]. Implementing and adopting evidence based falls prevention activities in the context of a RAC organisation requires embedding these activities in policy, processes and practices. To achieve this translation into practice systematic enquiry, synthesis and tailoring of falls prevention evidence for the local workplace is necessary [16–18]. Thus bringing people together with falls research expertise and local knowledge of barriers and facilitators to RAC workplace practices could facilitate effective translation of evidence into practice. One option to bring like-minded people together is a community of practice (CoP) that enables sharing of expertise and ideas, to innovate for change in pursuit of a common goal [19–21]. CoPs have been used in health care organisations with the intent of building capacity and improving health care outcomes with inconclusive results largely due to poor or absent evaluation. Improved impact evaluations are thus indicated [21–23]. A CoP was established to bring together RAC staff with an interest and goal in preventing falls with the intention of offering a social learning opportunity [19] and robustly evaluating its feasibility to facilitate translation of the current evidence using both objective outcomes and observed changes in health behaviour [24, 25]. The CoP was viewed as a complex intervention at the organisational level that could have differing impact across RAC facilities and the individual staff participating as members, dependent upon leadership, culture and staff behaviours [25–27]. Evaluation using this realist approach could identify how the CoP influenced the observed outcomes in different contexts of its membership, the RAC facility and RAC organisation [26, 28, 29].

Therefore the aims of this study were to evaluate the impact of a falls prevention CoP on its: i) membership; ii) actions at facility level; iii) actions at organisation level in translating falls prevention evidence into practice.

## Methods

### Design

**Fig. 1:**
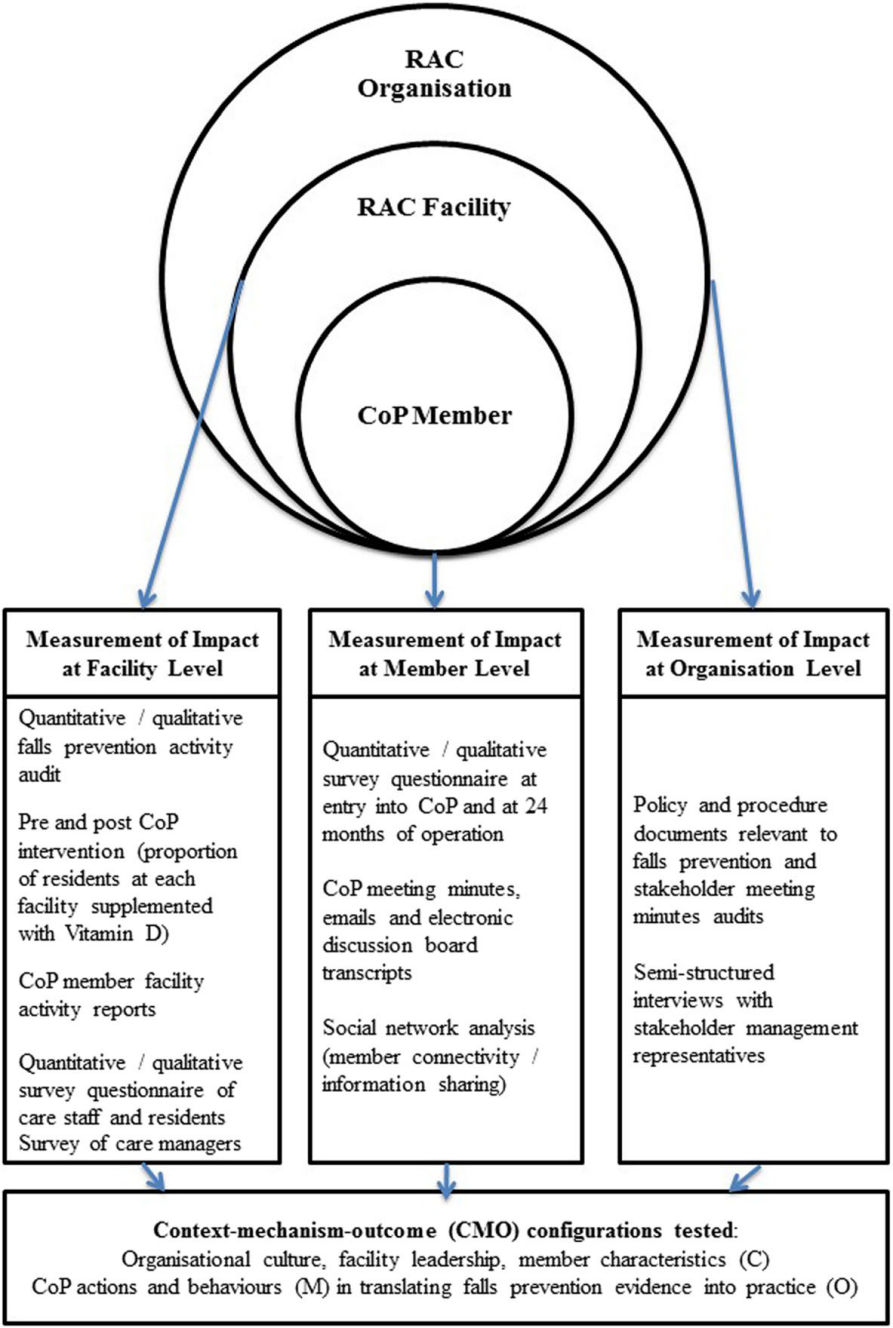
Overview of measuring CoP impact at member, facility and organisational level

This study used a convergent, parallel mixed methods evaluation design [30] based on a realist approach [31]. It formed part of a larger project to evaluate the impact of a falls prevention CoP on falls outcomes (including falls rates and injurious falls rates) in a RAC setting [32]. Briefly, realist approaches have been used when more than a description of an intervention’s outcomes is required; they seek in depth to identify how interventions trigger (mechanisms) the observed ‘outcomes’ in varying ‘contexts’ [26, 28, 31]. These triggers, termed ‘mechanisms’, are hidden causal factors that under certain conditions produce a particular outcome. Theoretical explanations of how a CoP might impact falls prevention were derived from the literature and stakeholder meetings using a context, mechanisms and outcomes (CMO) framework described elsewhere [32]. This framework was tested by posing the questions “what was it about the intervention that worked?”, “for whom?”, and “under what conditions?” Survey questionnaires, semi-structured interviews, observation journals, electronic transcripts, emails, meeting minutes, clinical records and policy documents provided data on CoP activity. An overview of the study design is shown in Fig. 1.

### Participants and setting

The RAC organisation was led by a Chief Executive Officer from a central administrative site. There were approximately 1200 full and part time care staff across 13 geographically diverse facilities providing mainly high level care in a home-like environment for 780 older people with a mean age of 84.01 years (SD = 4.56 years). Facilities were led by a care manager, with direct resident care provided mostly by care assistants supervised by professional nursing and allied health staff. The CoP partnered university researchers with staff across the 13 facilities. As the CoP was an intervention at organisation level and it’s actions interventions at facility level, all RAC facilities operated by the provider organisation were included. CoP membership was open to all RAC facility staff involved in resident care expressing an interest in falls prevention, following invitations delivered at facility and organisational levels. All facilities were represented by at least one CoP member with no more than 20 members at any one time for the duration of the study. Fourteen (65%) allied health professionals five (25%) care/deputy care managers and one (5%) researcher made up the membership. All CoP activities involving RAC staff or residents followed invitations provided at facility and organisation level yielding purposive samples. The study was conducted between November 2013 and November 2015.

### Outcome measures

The impact of the falls prevention CoP was evaluated at three levels; i) membership level (RAC staff who participated), ii) RAC facility level and iii) RAC organisation level on translating falls prevention evidence into practice, as shown in Fig. 1. This range of measured outcomes was used to inform theorised explanatory conjectured CMOs, which postulate how the outcomes were achieved considering the context in which they took place.

### Data collection and procedure

#### CoP member level

An online survey questionnaire was administered to CoP members via an email link using software by SurveyMonkey™ on entry into the CoP and following 24 months of CoP operation. Additional open response questions, modified from Ranmuthugala et al. [26], to determine experiences of CoP membership were included in the 24 months post CoP operation questionnaire. CoP electronic communication transcripts including emails and face to face meeting minutes were used for triangulation.

The researcher kept a journal to record her observations and reflections regarding CoP member participation and operation. The observations contributed to descriptions and explanations of CoP web based communication, activity and impact. Findings were presented to the CoP members to establish respondent validation or ‘member checking’ [30, 33].

The establishment of a community through connections between its members and knowledge flow through the community was recorded by counting postings on the CoP intranet discussion web page and whom the posting was shared with, in addition to members’ email frequency and attendance at eight face to face meetings. These CoP member interactions were recorded in a Microsoft Excel (2013) spreadsheet (Microsoft Corporation, Washington, USA).

#### RAC facility level

Measurement of the impact of the CoP at facility level prioritised improving the proportion of residents supplemented with vitamin D and development of falls prevention education. These priority areas were determined in the early phase of CoP operation when the CoP conducted an audit of falls prevention activity [34]. One of the 13 RAC sites did not participate in this intervention evaluation as it converted to a short term transition care facility and thus residents were not present for the duration of the intervention.

The proportion of residents at each facility supplemented with vitamin D was calculated from medication charts. Electronic dispensing records from supplying pharmacists were sourced to verify the accuracy of medication chart audits.

Surveys of care staff and residents were planned to scope what they knew and thought about falls and falls prevention to inform subsequent education program design.

Care staff consenting to participate were surveyed using a self-administered questionnaire distributed in a paper format at facility shift handovers, as computer access was limited. Explanation on completing the questionnaire was provided verbally and in written format by the shift registered nurse and the survey collection box was given prominence at the nurses’ station. Completed questionnaires were collected after two weeks by the researcher.

Consenting residents who did not have a diagnosis of cognitive impairment were surveyed face to face by a trained research assistant who read them the questions and recorded their responses.

All facility care managers (*n* = 13) were surveyed using an emailed short questionnaire modified from Ranmuthugala et al. [26] to determine their perception of CoP impact at their RAC facility following 24 months of CoP operation.

#### RAC organisation level

Policy manuals, procedure documents (including forms) and stakeholder meeting minutes were scrutinised by facility CoP members and professional staff at facilities during the falls prevention activity audit that is reported elsewhere [34]. Semi-structured interviews were conducted with two managerial representatives from the organisation, who had a role in overseeing the CoP project from its inception, using CoP evaluation questions modified from Ranmuthugala et al. [26]. The interviews were audio recorded using a digital dictaphone and followed the procedure recommended by Liamputtong [35]; face to face contact was established, the researcher chatted with the participants ensuring their comfort and gave an explanation of the interview procedure and recording process. Participants were encouraged to speak freely and on completion these conversations were transcribed verbatim by the researcher and checked by a second researcher for accuracy. Transcripts were returned to participants for member checking.

### Data analysis

#### Member level

CoP member pre and post questionnaire responses addressing capability, confidence, opportunity and motivation to champion falls prevention activity were extracted into SPSS version 22 software package (IBM SPSS Inc., Chicago IL, USA) and summarised using descriptive statistics. Differences pre CoP and 24 months post CoP operation were examined using a Wilcoxon signed rank test. Social Network Analysis enables the study of social processes by examining connections between individuals and communities [36]. As learning and knowledge exchange in CoPs is considered to occur at a social level, we undertook a social network analysis to determine the relationships and connections established between CoP members and patterns of knowledge flow within the CoP, that reflected learning. Data were organised in an excel matrix prior to entry into Ucinet 6 for Windows (Software for Social Network Analysis. Harvard, MA: Analytic Technologies). Exchanges between groups of members on the CoP discussion board provided frequency counts that were displayed in a matrix representing CoP member activity and connectivity. Qualitative data from CoP surveys, CoP face to face meeting minutes, researcher journal observations and emails were collected, transcribed verbatim and managed using NVivo analysis software (QSR International Pty Ltd. Version 10, 2012). Two independent researchers (JFC, AMH) read through all transcripts several times to become familiar with the data [37]. Correct responses in regard to falls prevention knowledge, were determined by two researchers with falls prevention expertise based on research evidence and best practice guidelines [13–15]. Where open question responses provided further categorical data frequency counts were also undertaken. Transcripts were analysed using deductive content analysis, which uses previous knowledge around the research topic, when a theory is being tested [38]. Question led category matrices were constructed [38] for member level responses based on the theoretical framework of what CoP activities or behaviours may have triggered the observed outcomes [26, 32]. As it was theorised CoP outcomes would be influenced by CoP member actions and behaviours the determinants of health behaviour change; capability, opportunity and motivation (to enact falls prevention behaviours) were used as a framework [24, 25]. Coding was thus framed around these behaviour change determinants of capability, opportunity and motivation [24] to explain what worked or didn’t work (CoP falls prevention actions, behaviours) for whom (members, RAC facilities, RAC organisation) and under what conditions [26, 29].

#### Facility level

Pre and post CoP audit measures for the proportion of residents per RAC facility on Vitamin D supplementation were described using proportion and percentage. Proportion differences pre and post intervention were examined using a dependent t-test or the non-parametric alternative Wilcoxon signed rank test. Cross-sectional quantitative survey responses from care managers, care staff and resident surveys were entered into SPSS version 22 software package (IBM SPSS Inc., Chicago IL, USA) and summarised using descriptive statistics. Qualitative care manager perceptions of CoP impact at their facilities were analysed using deductive content analysis and a capability, opportunity and motivation (to perform the behaviour) categorisation matrix as described previously [38].

#### Organisation level

Content analysis of falls prevention related policy and process documents (electronic and paper) together with management meeting minutes at baseline and following 24 months of CoP operation was undertaken to identify newly implemented falls related documents or process reporting. Data from semi-structured interviews of two management representatives was transcribed verbatim and analysed as described for CoP members**.**

#### Identification of causal mechanisms

After analyses for each level were completed, results from all 3 levels of measurement were examined to deduce what worked for whom and under what conditions; forming conjectured CMOs.

## Results

The impact of the falls prevention CoP at member, facility and organisation level is summarised in Table 1.

**Table 1:** Summary of CoP impact at member, facility and organisation level

Impact at member level	Impact at facility level	Impact at organisation level
Increased falls prevention knowledge	Annual evidenced-based falls prevention activity audit with intermittent spot checks	Falls policy (re-written and implemented)
Increased self-reported confidence and motivation to engage in falls prevention actions	Increased proportion of residents supplemented with vitamin D at all sites	Standardised fall definition adopted
Increased connections and collaborations with interdisciplinary CoP members	Falls prevention CoP listed as agenda item at facility staff meetings	New falls risk assessment tool placed in online assessment system
	Falls prevention committee formed	Aligned falls prevention management plan (developed and implemented)
	Falls prevention checklists for individual residents at highest risk of falling (“catch a falling star” program)	CoP newsletter (developed and implemented) 4 editions published
	Surveyed frontline care staff and residents to determine falls prevention education needs and preferences	Falls prevention CoP listed as agenda item at RAC Board Committee meetings
	Surveyed care managers to determine their perception of CoP impact at their site	
	Falls prevention poster checklist for staff and residents	
	Screening for safer resident footwear, clothing and lighting (night time sensor lights)	

### Member level impact

A total of 22 staff participated as CoP members for varying durations throughout the study, with 18 completing surveys pre CoP and 24 months post CoP operation.

#### Capability, opportunity and motivation to prevent falls

The greatest benefit of CoP membership reported by participants was improved evidence based falls prevention awareness and knowledge, Participating CoP member (P)11*“I’ve a better scope of knowledge relating to falls, the awful consequences and the evidence too.”* CoP members (*n* = 18) identified falls prevention strategies they were aware of at baseline [125 correct responses, median number of correct responses = 6.00 (IQR = 3–15)] and 24 months post CoP operation [221 correct responses, median number of correct responses = 10.50 (IQR = 4–28)]). There was a significant difference between the pre and post scores with post survey scores showing increases in knowledge [*p* < 0.001]. For example there was awareness of intrinsic risk factors like medication review*,* P6*“it’s improved my personal knowledge of falls management (multifactorial approach),”* P8*“I didn’t know the impact vitamin D and medications can have on falls until I joined the CoP. I bring this up when discussing with residents and staff.”*

When member survey responses regarding motivation and confidence to lead falls prevention activities were compared pre CoP and 24 months post CoP operation there were no significant differences (see Additional file 1). However when interviewed six members reported they felt motivated to attend external falls prevention events since joining the CoP, P3*“I’ve registered for the local falls conference,”* and eight became new contributors to facility falls prevention meetings, P9*“I’m part of a regular falls meeting at my facility now.”*

#### Connectivity amongst the membership

**Fig. 2:**
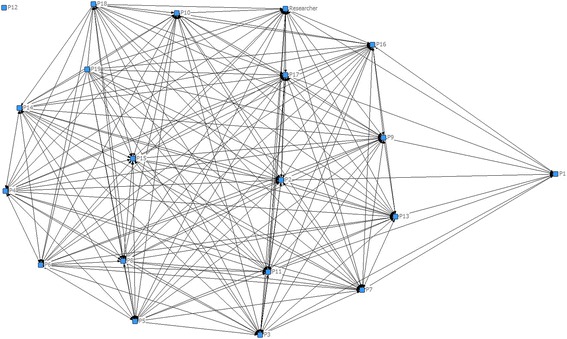
CoP member connectivity and knowledge flow amongst the membership

New or improved social connections were enabled, P7 *“it was great to get to know more staff”* and the opportunity to network, ask questions and share ideas with interdisciplinary colleagues (*n* = 11) was perceived as a membership benefit. This was reported as particularly relevant for members who were new to the RAC organisation or novice practitioners, P13*“It was lovely to have a place where I could ask questions,”* P9*“I feel I can contribute more to preventing falls and discussions about falls.”* Knowledge flow through the CoP and connections amongst members was evident through frequency counts of discussion board participation and post sharing amongst CoP members (see Additional file 2) and is represented visually in Fig. 2.

There were 11 different CoP web-based discussion topics supported by eight face to face meetings across the 24 months of CoP operation. Discussion topics included falls prevention auditing (29 member posts), promoting vitamin D supplementation (20 member posts), “Ask the CoP?” (21 member posts) and psychotropic medication use (11 member posts). The researcher and facilitator were the most connected across the entire membership providing a link between the research institution and RAC organisation. Six CoP members, who were therapists, became the most connected sharing more than eight postings and additional monthly email contact. Seventeen members shared falls prevention knowledge from the CoP with staff at their facilities, P7 *“I gave feedback at staff meetings, clinical meetings and shift handovers”* and ten with residents at their facilities, P8 *“we’ve discussed falls prevention in our new ‘Better Balance’ program.”*

The key barrier to member participation in the CoP was perceived to be lack of dedicated time due to competing interests P9 *“finding the time with so many other things to do,”* Manager 1 *“staff got no additional time to support involvement in the CoP, this was a barrier to getting things done.”*

### Facility level impact

#### Evidence-based falls prevention audit

The CoP was able to successfully lead and conduct a falls prevention activity audit at all 13 facilities in the organisation. The full procedure and results of the audit are described elsewhere [34].

#### Vitamin D supplementation

Significant improvements were made across 12 participating RAC facilities in the proportion of residents supplemented with vitamin D from July 2014 (baseline CoP audit) to November 2015 (follow up audit) with the mean increase in the proportion of residents receiving supplementation of 28.23% [(95%CI:15.96–40.51), *p* = 0.002] (see Additional file 3). P8*“we have printed out all the articles on vitamin D and the nursing staff have put the articles in all our visiting GP’s files and they discuss it with them so residents can be put on vitamin D.”* The key barrier to supplementation was identified as lack of some GP’s willingness to prescribe, P8 “*Some GPs are very resistive to any suggestions, it’s like they think what do you know?”*

#### Falls prevention education

To inform development of education, surveys of care staff and residents were undertaken to determine what they knew and thought about falls prevention following piloting at a single facility. Briefly, 147 care staff from eight facilities participated (response rate 37.9%); reminders to carry out falls prevention strategies by displaying posters around the facility were the most popular education preference [*n* = 80 (54.4%)]. Forty residents who did not have a diagnosis of cognitive impairment (response rate 83.3%) across six facilities participated in the resident survey. Education preferences included having a reminder poster for their room [*n* = 11 (27.5%)]. These findings led to the development and implementation of a pictorial falls prevention poster checklist across all facilities. One CoP member developed the ‘Catch a Falling Star’ program targeting residents assessed as at higher risk of falling and recurrent fallers using a personalised strategy checklist, P16*“we have the falling stars program, our residents have personal checklists to remind staff of the strategies to use at all times.”* Twenty two (78.6%) care staff participating in the survey from this facility discussed using the program when questioned about their knowledge of falls prevention strategies, Care staff 4*“I check and report on the falling star plans every shift,”* Care staff 11*“falling star plan says to always make sure they (resident) have their call bell in reach.”* Following CoP information sharing this program was then implemented by two additional facilities. Feedback from 12 care managers regarding CoP impact at their facility was strongly perceived to be: improved staff falls prevention awareness and actions through education and resources provided by the CoP members, Manager 9 *“given staff ideas on how to keep residents from falling, it’s a very precious tool.”*

#### Falls meetings, screening personal items and equipment provision

Falls prevention practices deemed to be effective at some facilities were shared with others for adoption, these included monthly facility “falls meetings” (*n* = 3) and falls prevention becoming an agenda item at staff meetings (*n* = 7), P3 *“we prioritised it, we discussed prevention together in team meetings to help them (staff) understand,” P2 “we helped staff realise how important it is by showing them the facts (displaying monthly falls rates)”* and screening resident footwear and clothing (*n* = 3) P16*“we went through the cupboards checking all items that were unsafe so family could remove, if it’s not there staff can’t put it on.”* Additional equipment, namely sensor lights for night-time toileting and bed or chair alarms, was introduced at two facilities.

#### Barriers to implementation

Barriers identified by CoP members to implementing fall prevention strategies included perceived lack of management support in realising the importance of prioritising falls prevention and member participation*,* P16*“there were some care managers who didn’t provide the project with the same importance as mine,”* P17*“at a facility where the manager is not committed, sees it (CoP) as less relevant, then it’s hard to get any impact,”* Manager 1*“if you’ve got care manager support then it’s front and centre in peoples’ minds.”*

### Organisation level impact.

#### Falls prevention policy and processes

CoP auditing of relevant falls related policy and process documents and management meeting minutes identified gaps in governance for targeted attention, Manager 1*“having a culture of wanting to improve is fundamental, acknowledge you are not perfect, have a willingness to change.”* A standardised fall definition to assist in clarifying the reporting of falls was adopted, “an unexpected event in which a person comes to rest on the ground or lower level” [39], P2*“the wording is easier for everyone to understand in this one,”* P5*“after discussing this and watching the simulation video I realised that some incidences should have been counted as falls at our facility.”* The drafting and completion of a falls prevention policy, risk assessment tool and aligned management strategies by the CoP was an iterative extensive process over 11 months, which engaged CoP members with RAC management. This reflected a cultural shift by both CoP members and RAC managers in their approach to falls from one of reactively managing falls to more proactive prevention, Manager 1*“there were gaps and I knew we didn’t have a standardised way of addressing falls, now we do all that proactive preventative stuff.”* The CoP liaised with clinical and management groups across the organisation through face to face and email discussions regarding content together with IT personnel for adaptation into workable electronic formats, Manager 1*“for me the major achievements of the CoP have been the policies and procedures, that was our gap and now I feel like we’re getting there.”*

#### Dissemination of CoP actions

Raising awareness and providing education regarding falls and falls prevention was addressed via a CoP newsletter in electronic and paper formats four monthly across the organisation to all levels of management, clinical working groups and staff, Manager 2*“it’s had a positive impact, I’ve seen it at facilities on coffee tables and noticeboards and heard staff talking about it.”* Ten care managers reported the CoP newsletter was distributed at their facilities and 11 thought it was a useful resource. The awareness of the problem of falls and importance of falls prevention raised by the CoP led to CoP reporting becoming an agenda item at the organisation’s Board Care Committees’ meetings, Manager 1*“it’s (newsletter) included in reports to the board care committees so they’ve got it as a standing agenda item.”*

#### Barriers to evidence translation

Barriers to the CoP translating evidence into practice from an organisational perspective were conflicting priorities and realising commitment in supporting dedicated staff time, Manager 2*“there was a lack of focus (on falls prevention), we didn’t give it dedicated time, but there are so many things we are involved in.”*

#### What worked? for whom? and under what conditions?

Results from each of the three levels of evaluation in the form of conjectured CMOs are presented in Table 2.

**Table 2:** Conjectured context-mechanism-outcome configurations

Member Level	
CCMO 1	Members who demonstrated higher levels of falls prevention knowledge and awareness (psychological capability) and felt they needed to action fall prevention strategies enough (reflective motivation), better engaged with other site staff to enable implementation of falls prevention strategies
CCMO 2	Members who participated more in CoP social learning opportunities, connected to experts, gained confidence and credibility and were motivated to make a greater contribution to falls prevention change at their facility
CCMO 3	Membership of a CoP enabled new and more frequent interdisciplinary connections to develop serving as a resource for guidance and reduced professional isolation within the organisation, when time to participate was supported by facility managers
RAC facility level	
CCMO 4	Facility visiting GPs who related to RAC staff (particularly CoP members and Nurse Practitioners) as credible peers and advocated for the recommended evidence significantly improved their proportion of residents supplemented with vitamin D
CCMO 5	Falls prevention programs were best implemented and adopted by frontline staff when the resident’s prevention strategies were prompted in novel ways and documentation of strategy enactment was made accountable by care managers
CCMO 6	Higher levels of care manager support, through realisation and prioritisation for staff to participate as CoP members and action falls prevention at their facility, enabled the implementation of evidence based practices
RAC organisation level	
CCMO 7	Organisational acknowledgment of gaps in governance and recognition of the consequences of not taking a more preventative approach (reflective motivation) regarding falls management changed the cultural focus towards pro-action, following greater engagement with the CoP
CCMO 8	Failure to offer opportunity in terms of dedicated time commitment for CoP members to learn and engage in falls prevention activity above existing professional duties, limited implementation of falls prevention activities
CCMO 9	Receiving regular reports on the CoP’s falls prevention actions created a stronger feedback loop from frontline care to general management and assisted in focussing attention on falls prevention

*CCMO* conjectured context mechanism outcome, *GP* General Practitioner

The conjectured CMOs demonstrated how the variability observed in translating evidence into practice was influenced by the RAC context. For example, the level of facility care manager support for CoP member participation and action (context), through realising the need to prioritise falls prevention activities (mechanism), influenced the success of translating evidence into practice (outcome).

## Discussion

Overall, interdisciplinary staff perceived that they benefitted from participating in a falls prevention CoP and that the CoP was able to translate falls prevention evidence into practice in the context of their individual facility and the RAC organisation.

### Member level

#### Reflection and realisation (CCMO 1 & 2)

Our study found that all CoP members benefited from membership by improving their knowledge of RAC falls prevention strategies through association with experts, but translating this knowledge into practice showed varied levels of success. Although possessing the relevant knowledge is a foundation step in the translation process identified by other studies [16, 18], simply having more knowledge did not necessarily mean CoP members moved it into use at facilities as other factors were involved [18, 40]. Furthermore, translation appeared to be triggered by CoP members who fully understood the negative consequences of a resident fall, reflected and realised the importance of engaging their colleagues in actioning falls prevention strategies at their facility. Reflection and realising negative consequences are traits reported elsewhere as important in triggering health behaviour change [25, 41].

#### Opportunities, connections and credibility (CCMO 2 & 3)

Membership of the falls prevention CoP enabled clinicians to gain confidence and credibility, through connections to experts and identify themselves as role models. This motivated members to then step up and contribute to falls prevention change at their facilities, particularly if they were new to the field of falls prevention. Social learning opportunity is a characteristic of CoPs whereby association of novice with expert in a field can lead to professional identity building through sharing and collaborating [20, 26]. Higher levels of connectivity in social networks such as CoPs have been associated with a stronger sense of community and greater resource exchange amongst members [42, 43]. Membership of the CoP enabled new and more frequent interdisciplinary connections to develop which then served as a resource for guidance and reduced professional isolation within the organisation as identified by Ranmuthugala et al. [26].

### Facility level

#### Relationships, credibility and advocating (CCMO 4)

Improvement in the proportion of residents supplemented with vitamin D varied across the 12 participating facilities, which could have been influenced by the enabling or disenabling actions of the visiting GPs as the main prescribers (of medications). It was perceived by CoP members that GPs who viewed RAC staff as credible peers, regarding providing falls prevention evidence, advocated for vitamin D supplementation, whereas those who didn’t acted as a barrier. Other studies have found that doctor and nurse cooperation can influence the success of intervention implementation: A systematic review of interdisciplinary interventions in nursing home settings reported positive impacts on resident outcomes when the resident’s doctor participated in the intervention [44]. Conversely Steinmo et al. [45] also noted conflict between doctor and nurse was a key barrier to implementation success of a quality improvement program in a health care setting.

#### Sharing, motivation and reinforcement (CCMO 5)

More falls prevention activities were implemented at RAC facilities that had manager support and when CoP members were motivated and provided meaningful resources. For example the ‘Catch a Falling Star’ program, supported by the facility manager, was one CoP member’s motivational way of sharing falls prevention strategies that made sense to facility staff and resulted in uptake at their facility. Motivational ways of sharing knowledge is recommended for enabling knowledge transfer [20, 45]. Enactment of falls prevention strategies by frontline care staff was observed when facility managers supported staff accountability, through reinforcing documentation of their actions in resident notes. Reinforcement of desired health behaviours has been shown to assist in habit formation [24, 25].

#### Prioritising and supporting (CCMO 6)

CoP members who were given the time to attend face to face CoP meetings and became involved in web-based discussion and collaboration were more successful at implementing falls prevention evidence and practice change at their facility. This action was perceived by CoP members to be triggered when care manager’s realised dedicated time was needed for CoP members to lead falls prevention change and were able to prioritise support for CoP participation. For example, supported CoP members implemented additional multifactorial falls prevention strategies such as tailored resident falls prevention plans, footwear screening and facility committee meetings. Conversely at facilities where CoP members were not supported to participate in CoP meetings and discussions there was limited implementation of evidence based practices. Limited dedicated time for staff to be involved was a common barrier reported in other health implementation studies [27, 45, 46].

### Organisation level

#### Acknowledgment, engagement and cultural change (CCMO 7)

The CoP, were able to identify a gap in the falls management policy and procedures. The CoP engaged management using information on the pros and cons of taking preventative action to gain their support in a cultural change to approaching falls. Taking a more proactive cultural approach to falls may lead to better outcomes for residents as RAC culture has been linked to quality outcomes for residents [27, 47]. Providing information on the pros and cons of performing a behaviour has been used to facilitate health behaviour change [24]. The further engagement of CoP members, who were clinical staff delivering resident care, in writing the new falls policy and procedures brought authenticity and relevancy. This tailoring of knowledge by the users has been identified as a step in successful evidence translation [18, 21].

#### Opportunity and engagement (CCMO 8)

At an organisational level failure to consistently support opportunity, in terms of dedicated time commitment, for CoP members to learn and engage in falls prevention activity was perceived to limit implementation of falls prevention activities. Whilst CoP members were cognisant of the fact that the organisation had to manage a range of complex issues, they felt this still reflected a lack of realised importance of the need to learn and action falls prevention in the workplace and achieve even better outcomes. Limited time and resources was identified in other studies as a barrier to work place learning and implementing new practices [27, 48].

#### Feedback loop and focus (CCMO 9)

Regular CoP reporting on their falls prevention actions and outcomes, created a strong feedback loop from frontline care staff to organisational management. Recognition of higher levels of feedback for systems, teams or individuals is a factor linked with successful implementation [21, 49] and use of evidence in practice [16]. CoP reporting to the organisation’s Board Committees assisted in focussing attention and subsequent support for falls prevention activity. Organisational support has been reported as a CoP enabling mechanism by Ranmuthugala et al. [26] whilst shifting organisational priorities has been identified as a barrier to implementation by others [27, 50].

### Limitations

Evaluation and explanation of the impact of operating a falls prevention CoP on falls rates and injurious falls rates was beyond the scope of this study and these findings are reported elsewhere [51]. In this study we have postulated possible mechanisms that triggered the observed outcomes under certain contextual conditions. Whilst findings from evaluating a single RAC organisation are not generalizable they provide valuable learnings for similar RAC organisations looking to translate falls evidence into practice. The size of the CoP may appear small (*n* = 20) but we feel it reflects the authentic number of staff a RAC organisation of this size may assign to participate in a given project. Whilst elements of this study relied on self-report, we have supported validity and credibility of the findings by incorporating quantitative data where possible, triangulating findings using multiple data sources and maintaining an audit trail. Ideally interviews of care staff, facility managers and representatives of the organisation’s Board Committees would have provided further depth to our insights however the pragmatics of such an undertaking were beyond the scope of this study. The intranet software was unable to track members accessing the CoP web site unless they posted comments on the electronic discussion board but future upgrades to the software should have the capacity to track access across all areas.

## Conclusions

An interdisciplinary falls prevention CoP provided connections and knowledge gains amongst its members and was able to facilitate translation of falls prevention evidence into practice in the context of a RAC facility and RAC organisation. Translation worked best at facilities with an active CoP member connected to evidence with management support in a proactive falls prevention culture. Support by RAC management to provide the necessary investment in staff time to better enable change in falls prevention practice is essential for success. Future research should continue to test these conjectured mechanisms of action noting the contextual conditions that produce the desired or undesired outcomes. This may better inform how CoPs impact their membership and the translation of evidence into practice.

## Additional files

Additional file 1: CoP member confidence, motivation and opportunity to engage in intranet usage and lead falls prevention activity. (DOCX 15 kb)

Additional file 2: Matrix of knowledge flow through CoP and member connections through discussion board participation. (DOCX 21 kb)

Additional file 3: Proportion of residents supplemented with vitamin D measured in July 2014 and re-measured November 2015. (JPEG 48 kb)

## Abbreviations

CMO: Context-Mechanism-Outcome
CoP: Community of Practice
P: Participating CoP member
RAC: Residential Aged Care
