# Supplementary material for: Evaluating the impact of a falls prevention community of practice in a residential aged care setting: a realist approach
Source: BMC Health Serv Res. 2018 Jan 15;18:21. doi: 10.1186/s12913-017-2790-2 (PMC5769423; doi:10.1186/s12913-017-2790-2)
Supplement: Supplementary file 1 — CoP member confidence, motivation and opportunity to engage in intranet usage and lead falls prevention activity. (DOCX 15 kb) [file 12913_2017_2790_MOESM1_ESM.docx]

Additional file 1 CoP member confidence, motivation and opportunity to engage in intranet usage and lead falls prevention activity

| Item | SA | A | U | D | SD | No Response | *Pre Median(IQR) / Post Median(IQR) | p value |
| --- | --- | --- | --- | --- | --- | --- | --- | --- |
|  | *Pre / Post | *Pre / Post | *Pre / Post | *Pre / Post | *Pre / Post | *Pre / Post |  |  |
| I use the intranet as part of my everyday work practice | 9/7 | 7/10 | 0/1 | 2/0 | 0/0 | 0/0 | 4.5(2-5)/4(3-5) | 0.957 |
| I have easy access to the intranet at my RAC site | 11//11 | 6/7 | 0/0 | 1/0 | 0/0 | 0/0 | 5(2-5)/5(4-5) | 0.480 |
| I am confident using the intranet for communication with CoP members | 5/6 | 12/11 | 0/1 | 0/0 | 1/0 | 0/0 | 4(1-5)/4(3-5) | 0.564 |
| I have time to use the intranet at my work site for CoP participation | 3/2 | 10/6 | 3/7 | 1/3 | 1/0 | 0/0 | 4(1-5)/3(2-5) | 0.190 |
| I feel confident using the intranet discussion board with CoP members | 2/4 | 9/9 | 5/2 | 2/3 | 0/0 | 0/0 | 4(2-5)/4(2-5) | 0.589 |
| I am regularly informed of falls outcomes at my RAC site | 6/6 | 7/8 | 2/3 | 2/1 | 1/0 | 0/0 | 4(1-5/4(2-5) | 0.317 |
| I feel motivated to be a falls champion at my RAC site | 3/6 | 10/5 | 3/6 | 1/1 | 1/0 | 0/0 | 4(1-5/4(2-5) | 0.763 |
| I feel confident to be a falls champion at my RAC site | 2/4 | 11/7 | 2/6 | 0/1 | 3/0 | 0/0 | 4(1-5/4(2-5) | 0.305 |
| SA Strongly Agree, A Agree, U undecided, D Disagree, SD Strongly Disagree, CoP Community of Practice, RAC Residential Aged Care | | | | | | | | |
| *Pre CoP membership / 24 months Post CoP operation | | | | | | | | |
|  | | | | | | | | |
