# Supplementary material for: Evaluating the impact of a falls prevention community of practice in a residential aged care setting: a realist approach
Source: BMC Health Serv Res. 2018 Jan 15;18:21. doi: 10.1186/s12913-017-2790-2 (PMC5769423; doi:10.1186/s12913-017-2790-2)
Supplement: Supplementary file 2 — Matrix of knowledge flow through CoP and member connections through discussion board participation. (DOCX 21 kb) [file 12913_2017_2790_MOESM2_ESM.docx]

Additional file 2 Matrix of knowledge flow through CoP and member connections through discussion board participation

|  | P1 | P2 | P3 | P4 | P5 | P6 | P7 | P8 | P9 | P10 | P11 | P12 | P13 | P14 | P15 | P16 | P17 | Res | P18 | P19 |
| --- | --- | --- | --- | --- | --- | --- | --- | --- | --- | --- | --- | --- | --- | --- | --- | --- | --- | --- | --- | --- |
| P1 |  | 1 | 1 | 0 | 0 | 0 | 1 | 0 | 1 | 0 | 1 | 0 | 1 | 0 | 0 | 1 | 1 | 1 | 0 | 0 |
| P2 | 1 |  | 5 | 5 | 5 | 3 | 6 | 4 | 6 | 2 | 6 | 0 | 5 | 2 | 2 | 6 | 6 | 5 | 0 | 0 |
| P3 | 1 | 4 |  | 4 | 4 | 6 | 8 | 6 | 7 | 2 | 7 | 0 | 7 | 2 | 2 | 9 | 9 | 9 | 0 | 0 |
| P4 | 0 | 4 | 4 |  | 4 | 3 | 4 | 3 | 3 | 1 | 3 | 0 | 3 | 1 | 1 | 5 | 5 | 4 | 0 | 0 |
| P5 | 0 | 4 | 5 | 4 |  | 3 | 6 | 3 | 3 | 2 | 3 | 0 | 3 | 2 | 2 | 6 | 6 | 5 | 0 | 0 |
| P6 | 0 | 3 | 4 | 4 | 3 |  | 6 | 4 | 3 | 2 | 3 | 0 | 3 | 2 | 2 | 8 | 8 | 7 | 0 | 0 |
| P7 | 1 | 4 | 5 | 3 | 3 | 5 |  | 4 | 5 | 1 | 5 | 0 | 5 | 1 | 2 | 8 | 8 | 7 | 1 | 0 |
| P8 | 0 | 4 | 6 | 4 | 4 | 5 | 6 |  | 6 | 1 | 6 | 0 | 6 | 1 | 1 | 8 | 8 | 8 | 0 | 0 |
| P9 | 1 | 5 | 5 | 3 | 3 | 3 | 5 | 4 |  | 2 | 6 | 0 | 5 | 2 | 2 | 5 | 5 | 5 | 0 | 0 |
| P10 | 0 | 1 | 1 | 1 | 1 | 1 | 1 | 1 | 1 |  | 1 | 0 | 1 | 1 | 1 | 1 | 1 | 1 | 0 | 0 |
| P11 | 1 | 7 | 6 | 3 | 3 | 3 | 6 | 5 | 9 | 1 |  | 0 | 6 | 1 | 1 | 6 | 6 | 6 | 0 | 0 |
| P12 | 0 | 0 | 0 | 0 | 0 | 0 | 0 | 0 | 0 | 0 | 0 |  | 0 | 0 | 0 | 0 | 0 | 0 | 0 | 0 |
| P13 | 1 | 3 | 4 | 2 | 2 | 2 | 4 | 3 | 4 | 1 | 4 | 0 |  | 1 | 1 | 4 | 4 | 4 | 0 | 0 |
| P14 | 0 | 1 | 1 | 1 | 1 | 1 | 1 | 1 | 1 | 1 | 1 | 0 | 1 |  | 1 | 1 | 1 | 1 | 0 | 0 |
| P15 | 0 | 1 | 1 | 1 | 1 | 1 | 2 | 1 | 1 | 1 | 1 | 0 | 1 | 1 |  | 2 | 2 | 2 | 1 | 0 |
| P16 | 1 | 7 | 11 | 8 | 6 | 9 | 12 | 8 | 8 | 2 | 8 | 0 | 8 | 2 | 3 |  | 15 | 14 | 1 | 0 |
| P17 | 4 | 9 | 13 | 7 | 6 | 9 | 15 | 7 | 10 | 2 | 10 | 0 | 10 | 2 | 4 | 18 |  | 17 | 1 | 0 |
| Res | 4 | 19 | 32 | 21 | 17 | 21 | 31 | 22 | 24 | 5 | 24 | 0 | 24 | 5 | 7 | 39 | 39 |  | 1 | 0 |
| P18 | 0 | 1 | 1 | 1 | 1 | 1 | 3 | 1 | 1 | 1 | 1 | 0 | 1 | 1 | 3 | 3 | 3 | 3 |  | 0 |
| P19 | 0 | 1 | 1 | 1 | 1 | 1 | 1 | 1 | 1 | 1 | 1 | 0 | 1 | 1 | 1 | 1 | 1 | 1 | 1 |  |

P=Participating CoP member, Res=Researcher
