# Supplementary figures and images for: Evaluating the impact of a falls prevention community of practice in a residential aged care setting: a realist approach
Source: BMC Health Serv Res. 2018 Jan 15;18:21. doi: 10.1186/s12913-017-2790-2 (PMC5769423; doi:10.1186/s12913-017-2790-2)

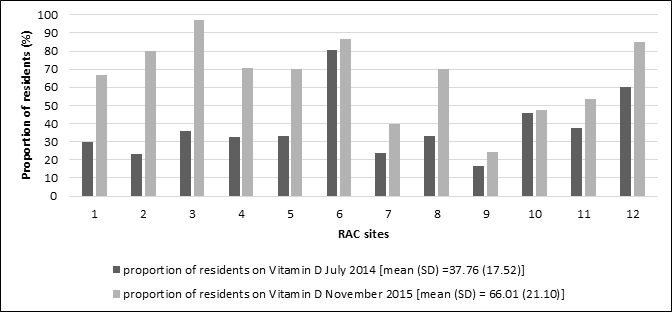

Supplement: Supplementary file 3 — Proportion of residents supplemented with vitamin D measured in July 2014 and re-measured November 2015. (JPEG 48 kb) [file 12913_2017_2790_MOESM3_ESM.jpg]
